# Ultrasonographic Confirmation of Nasogastric Tube Placement in the COVID-19 Era

**DOI:** 10.3390/jpm12030337

**Published:** 2022-02-23

**Authors:** Vasiliki Tsolaki, George E. Zakynthinos, Paris Zygoulis, Fotini Bardaka, Aikaterini Malita, Vasileios Aslanidis, Epaminondas Zakynthinos, Demosthenes Makris

**Affiliations:** Critical Care Department, University Hospital of Larissa, Faculty of Medicine, University of Thessaly, Mezourlo, 41335 Larissa, Greece; gzakynthinos2@gmail.com (G.E.Z.); paris.zygoulis@gmail.com (P.Z.); bardakafotini@yahoo.gr (F.B.); kat.malita@gmail.com (A.M.); vasaslan21@gmail.com (V.A.); ezakynth@yahoo.com (E.Z.); appollon7@hotmail.com (D.M.)

**Keywords:** nasogastric tube, POCUS, ultrasonography, intensive care unit

## Abstract

Background: Nasogastric tube (NGT) placement is a daily routine in the Intensive Care Unit (ICU), and misplacement of the NGT can cause serious complications. In COVID-19 ARDS patients, proning has emerged the need for frequent NGT re-evaluations. The gold standard technique, chest X-ray, is not always feasible. In the present study we report our experience with the use of ultrasonographic confirmation of NGT position. Methods: A prospective study in 276 COVID-19 ARDS patients admitted after intubation in the ICU. Ultrasonographic evaluation was performed using longitudinal or sagittal epigastric views. Examinations were performed during the initial NGT placement and every time the patients returned to the supine position after they had been proned or whenever critical care physicians or nurses considered that reconfirmation was necessary. Results: Ultrasonographic confirmation of correct NGT placement was feasible in 246/276 (89.13%) patients upon ICU admission. In 189/246 (76.8%) the tube could be visualized in the stomach (two parallel lines), in 172/246 (69.9%) the ultrasonographic whoosh test (“flash” due to air instillation through the tube, seen with ultrasonography) was evident, while in 164/246 (66.7%) both tests confirmed correct NGT placement. During ICU stay 590 ultrasonographic NGT evaluations were performed, and in 462 (78.14%) cases correct NGT placement were confirmed. In 392 cases, a chest X-ray was also ordered. The sensitivity of ultrasonographic NGT confirmation in these cases was 98.9%, specificity 57.9%, PPV 96.2%, and NPV 3.8%. The time for the full evaluation was 3.8 ± 3.4 min. Conclusion: Ultrasonographic confirmation of correct NGT placement is feasible in the initial placement, but also whenever needed thereafter, especially in the COVID-19 era, when changes in posture have become a daily practice in ARDS patients.

## 1. Introduction

Nasogastric tube (NGT) placement is performed in every patient hospitalized in the Intensive Care Unit (ICU) to facilitate enteral nutrition, administer medication, and for gastric decompression [1,2]. Misplacement of the NGT incidence varies from 0.5 to 89% and can cause serious complications such as aspiration pneumonia, empyema, pneumothorax, hemothorax, pneumomediastinum, or even intracranial insertion [2,3]. Confirmation of correct placement of the tube can be performed by auscultating air through the tube (whoosh test), observation of aspirated fluid, and pH measurement of the gastric aspirate. Yet the gold standard technique is abdominal X-ray confirmation combined with aspirate testing [3].

The reference pH value is different between the gastric and lung aspirate. Gastric pH ranges from 1 to 5.5, while lung aspirate is more alkaline. Gastric aspirate is only achieved in half of the patients, and the use of proton pump inhibitors, prolonged fasting, and enteral feeding may alter the values of gastric pH, complicating the results further [4]. Aspiration of alkaline fluid cannot exclude the presence of the tube in the distant esophagus. Auscultation, on the other hand, may be tricky when managing COVID-19 patients, as personal protective equipment may hinder the use of a stethoscope.

On the other hand, radiographies are not always easily available throughout the day, and certain delays in the initiation of enteral feeding may occur. In the COVID-19 era, a significant proportion of patients admitted in the ICU are placed in the prone position. Changes of patients’ posture (from supine to prone and vice versa) occur at least once a day (according to local protocols, every 16–30 h) [5]. Thus, the NGT position may have to be rechecked every day or every other day. Performance of daily abdominal X-rays is time and resource consuming, and it exposes patients to a certain degree of radiation.

Ultrasonographic NGT placement confirmation is increasingly being recognized as a safe, alternative technique, yet contradictory results from published studies, with mainly small numbers of included patients, have precluded the wide acceptance of the technique as a standard procedure [6]. Point Of Care Ultrasonography (POCUS) is gaining place in everyday clinical practice in ICU practitioners, as it is available at the bedside 24 h a day/7 days a week, and, as it is performed by clinicians caring for the patients, it can answer clinical questions [7]. Critical care nurses, untrained in the ultrasound technique, can easily gain skills in accurately performing and interpreting POCUS images, at the patient’s bedside [8]. In the present study, we report our experience in NGT confirmation using ultrasonography in COVID-19 ARDS, intubated, ICU patients.

## 2. Methods

This prospective study was conducted in an 18-bed, COVID-19 dedicated ICU, in a tertiary hospital in Central Greece. All COVID-19 mechanically ventilated, ARDS patients admitted from 2 April 2020 until 30 November 2021 were evaluated. Patients having undergone gastric surgery in the past were excluded from the analysis. The study was approved by the local ethics committee (University Hospital of Larissa) (55949/2020, date of approval: 23 December 2020), with a waiver for informed consent.

**Figure 1:**
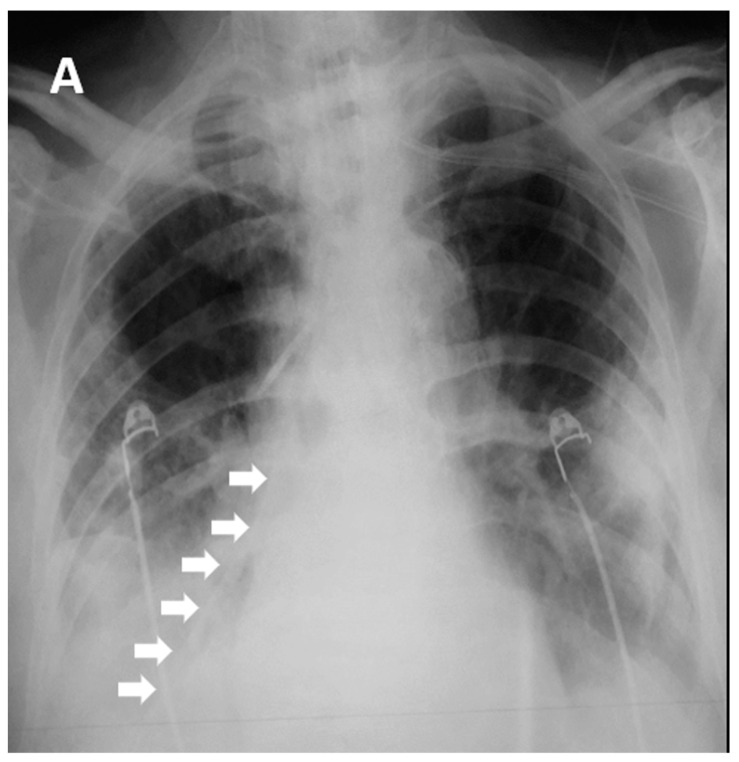
Chest X-ray in one of the first COVID-19 ARDS patients admitted in our ICU. The patient was turned from prone to the supine position on the 4th ICU day, late in the night, and nasogastric tube position was checked with palpation of a “flash” of air in the epigastrium, although there could not be observed any aspirated fluid. Enteral nutrition was started. Eight hours later the patient became hypoxemic, and increased tracheobronchial secretions were noted. A chest X-ray was ordered which revealed the NGT mispositioning in the right lower lobe. White arrows indicate the misplaced nasogastric tube in the Right Lower Lobe.

A nasogastric tube was placed in every patient with COVID-19 ARDS, upon ICU admission. Critical care physicians inserted the NGTs. Confirmation of correct NGT placement was performed with abdominal ultrasonography using a convex probe with low frequency (2–5 MHz). The probe was positioned in the sagittal and longitudinal epigastric section. Correct NGT placement was considered if either a single or double parallel lines of the tube were visualized in the gastric antrum or pylorus (Figure 1) and/or there was a dynamic appearance of air entering the stomach, instilled through the NGT (ultrasonographic whoosh test) (Videos S1 and S2). Confirmation was considered is cases in which both ultrasonographic tests were present or there was one ultrasonographic test and a positive “palpation test”. A positive “palpation test” was considered the test when a “flash” of air was palpated in the epigastrium, after installation of 50 mL of air through the NGT. When correct NGT placement could not be evaluated with one of the abovementioned criteria, an abdominal X-ray was ordered, and enteral nutrition was delayed until the radiograph was performed. Confirmation of correct NGT position was re-performed using ultrasonography every time the patients returned to the supine position after they had been proned, or whenever critical care physicians or nurses considered that reconfirmation was necessary.

### Statistical Analysis

Kolmogorov–Smirnov tests were applied to test the variable distribution. Continuous variables with normal distribution were reported as means and standard deviation (SD). Categorical variables were reported as numbers (*n*) and percentages (%). Statistical analyses were performed using SPSS version 26.0 (IBM Corporation, New Orchard Road, Armonk, NY, USA).

## 3. Results

**Figure 2:**
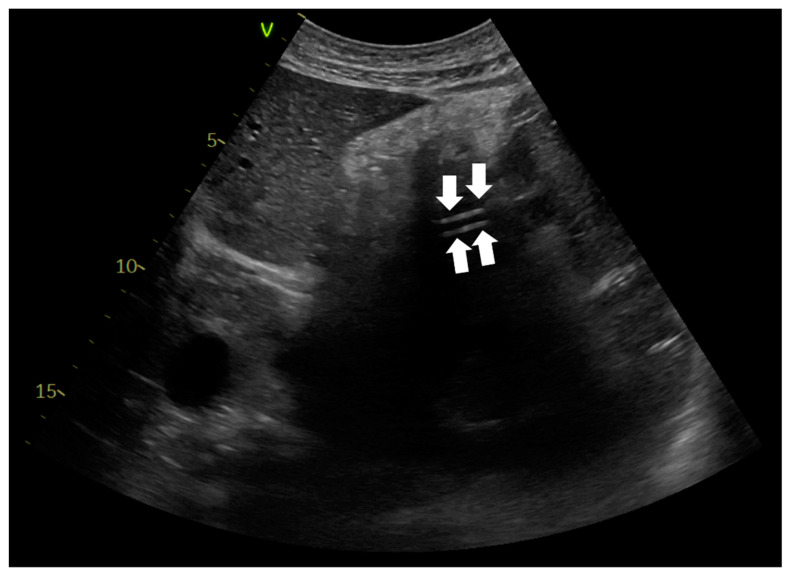
Abdominal ultrasonography. Ultrasonographic confirmation of nasogastric tube presence in the stomach. The liver is seen on the left of the image. Two parallel lines are noted, corresponding to the NGT (white arrows).

Two hundred and seventy-seven patients with COVID-19 ARDS were admitted in the ICU, and one was excluded from the analysis as he had undergone bariatric surgery 10 years previously (Figure 2, Table 1). All 282 patients, apart from three, had a NGT placed immediately after ICU admission. Those three patients were proned immediately after admission without a NGT, which was placed after they returned to the supine position. After the first six COVID-19 ARDS patients were admitted to the ICU, we faced one unfortunate NGT misplacement in a patient who turned to the supine position after he had been proned for sixteen hours. A chest X-ray was delayed, and enteral feeding was initiated after a “palpation” positive test. Eight hours later the patient became severely hypoxemic with increased secretions. Chest X-ray revealed that the NGT had been positioned in the right lower lung lobe (Figure 1). After that we decided to adopt a protocol of ultrasonographic confirmation of correct NGT position.

**Figure 3:**
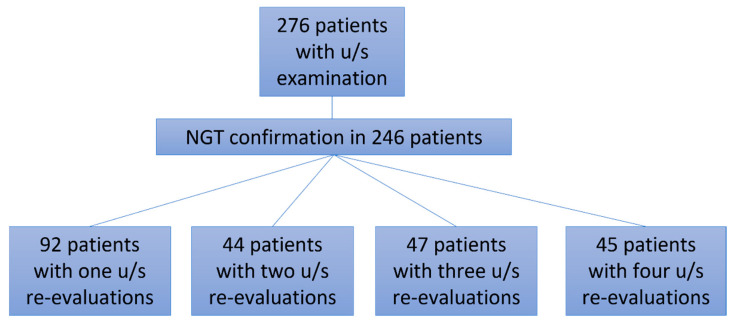
Flow chart of the patients with ultrasonographic confirmation of nasogastric tube. Flow chart of the patients in whom ultrasonographic NGT confirmation was performed. NGT, nasogastric tube; u/s, ultrasound.

**Figure 4:**
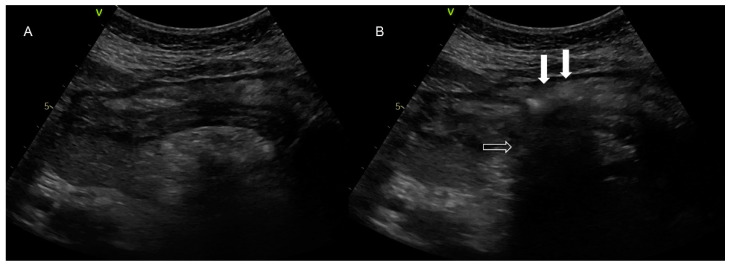
Abdominal ultrasonography. In picture (**A**), there is a presentation of the stomach at the center of the image in the longitudinal axis, while the hyperechoic structure beneath is pancreatic tissue. In picture (**B**), the image is obtained after instillation of a “flash” of air (white arrows) through the nasogastric tube. The pancreatic tissue is obscured (empty arrows), as an amount of air interferes between the ultrasonographic beam and the pancreas.

Ultrasonographic evaluation of NGT position was performed in 276 patients, and confirmation of correct NGT placement was feasible in 246/276 (89.13%) patients. In 189/246 (76.8%) the tube could be visualized in the stomach (Figure 3), in 172/246 (69.9%) the ultrasonographic whoosh test was evident (Figure 4, Videos S1 and S2), while in 164/246 (66.7%) both tests confirmed correct NGT placement. All the patients had additional confirmation with palpation of a “flash” of air in the epigastrium instilled through the NGT. In the patients in whom the NGT could not be visualized and an ultrasonographic whoosh test was negative, a chest X-ray was performed. In all patients the NGT was located in the stomach.

Five hundred ninety ultrasonographic evaluations, in total including an initial evaluation and re-evaluations, were performed during the study period. Four hundred sixty-two (78.3%) confirmations of correct NGT placement could be evaluated (Table 1, Figure 2). In the 128 cases (21.7%) where the NGT could not be visualized with the ultrasound, a chest X-ray was ordered. In ninety-eight patients the change in the patients’ position was performed after midnight, and NGT confirmation was performed palpating an air “flash” in the epigastrium, inserted through the NGT. In these patients, enteral nutrition was initiated. The NGT was evaluated as soon as possible, but these patients were not included in the analysis.

Chest X-rays to confirm NGT placement after every postural change were not ordered. On the contrary, 392 routine X-rays were performed in these patients. In 372 patients, correct NGT placement was identified with ultrasonography and confirmed with the X-ray. In one patient although there was an ultrasonographic image of the whoosh test, chest X-ray identified the NGT in the esophagus. In seven more, the NGT tube was visualized with both ultrasonographic methods, yet its position could not be verified with the X-ray. In four cases the NGT could not be identified by ultrasonography, but chest X-ray confirmed its presence in the stomach, and in eight patients the NGT was not visualized with u/s and chest X-ray did not identify it, so the tube had to be repositioned (sensitivity 98.9%, specificity 57.9%, positive predictive value 96.2%, negative predictive value 3.8%). In those patients with a positive ultrasonographic test and a negative X-ray, the NGT was not used for feeding; ultimately (after 5 h the longest) gastric fluid was aspirated, thus confirming correct NGT placement.

The time to evaluate the correct NGT placement (identification of the tube, ultrasound whoosh test) was 3.8 ± 3.4 min. The procedure was demonstrated in critical care nurses, and after five examinations, they could identify the NGT (ultrasonographic evaluation carried by a physician).

## 4. Discussion

In the present study we have shown that by using Point of Care Ultrasonography, nasogastric tube placement confirmation is feasible at the bedside, especially in times of crisis, when an increased number of patients overwhelm ICU bed and staff capacity. COVID-19 patients, requiring multiple postural changes, warranted frequent NGT position confirmations. Evaluation of the correct NGT placement was achieved in 89% of the patients when the NGT was initially inserted upon ICU admission, while it could be re-evaluated in 78% of the COVID-19 ARDS patients, after returning from the prone to the supine position. The examination was easy to perform, and it took less than five minutes to be completed.

Nasogastric tube placement is a routine clinical practice in ICUs, as enteral nutrition and medication administration depend on a NGT presence. The whoosh test is the simplest and most widely adopted test to confirm the correct placement of an NGT. Nevertheless, in the COVID-19 era, wearing personal protective equipment (PPE) has hindered many sensations in the clinical examination of the patients. Auscultation has become a real challenge, as hearing over PPEs is restricted. Moreover, auscultation with insufflation of air is unreliable, as it cannot detect most lung mispositions [9]. On the other hand, chest X-rays, the gold standard technique, are not always feasible. In busy ICUs with many patients, due to changing position from supine to prone and back, at different times during the day, it is not always feasible to order radiographs in order to confirm the correct NGT position and initiate enteral feeding. Thus, a more practical approach is needed.

Point of Care Ultrasonography has become a valuable tool in managing critical care patients. For decades, clinical ultrasound examinations have been performed by sonographers on cumbersome machines and interpreted by radiologists and cardiologists in dark rooms. With technological advances, a decrease in the size and cost of ultrasound machines has resulted in an expansion in the application of point-of-care ultrasound examination. This is ultrasound examination performed and interpreted by the clinician at the bedside, and POCUS has proven to be user friendly and quickly performed with recognizable, real-time findings [7]. Not only doctors but critical care nurses, with no experience in ultrasonography, can easily be trained in acquiring and interpreting images obtained with POCUS [8]. Recently, after only a four-hour training program, critical care nurses could accurately identify correct NGT placement and gastric residual volume [8]. We believe that critical care nurses with no experience in ultrasonography may be easily trained to identify NGT with POCUS, with a small learning curve.

In the present study we found that Point Of Care Ultrasonography can be used to evaluate correct NGT position in COVID-19 ARDS patients, in whom multiple changes in posture (supine–prone) during the day may cause frequent NGT dislodgments. During the COVID-19 pandemic, prone position has become a daily practice in hundreds of patients [5]. With these frequent changes in patients’ postures on a daily basis and at different time points, the need for frequent re-evaluations of correct NGT position has revealed the difficulties and unfeasibility of the radiographic reliance for NGT confirmation. Even auscultation is not always informative. Apart from being easily available at the bedside 24/7, ultrasonographic NGT confirmation prevents additional delays in re-initiation of the enteral feeding, when waiting for the chest X-ray to assure the NGT position. In the present study, ultrasonographic evaluation lasted less than five minutes in most of the cases. In a recent study using color doppler ultrasonography to detect the position of the NGT, it took significantly less time to confirm the NGT with ultrasound compared to the chest X-ray (10 min versus 90 min), confirming previous results [10,11].

In the present study, NGT confirmation could be evaluated in 89% of the patients during the initial placement of the tube, while concerning re-evaluations, correct placement was confirmed in 78% of these cases, while 98 patients were not tested with POCUS. When coupled with a chest X-ray, the sensitivity of ultrasonographic evaluation was 98.9%. This could be attributed to the lack of experienced personnel during the position changes, as not all the ICU staff were trained in POCUS, a finding signifying the need for u/s training of the critical care staff (physicians and nurses). Secondly, as the need of NGT re-evaluation emerged during the following days of ICU admission, gastroparesis may partially explain the lack of image acquisition.

The role of ultrasound confirmation of correct NGT placement has been contradictory. Ultrasonographic confirmation of correct NGT placement has been reported to present high sensitivity and specificity in studies conducted in a small number of patients in the prehospital setting [12], the emergency department [13,14], and the ICU [15,16]. Interestingly, Ye et al. proposed an algorithm, tested in 157 critically-ill patients, for ultrasonographic nasointestinal tube placement and reported a sensitivity of 96.4% and a specificity of 90% for the method [17]. Tsujimoto conducted a Cochrane Database Systematic Review of ultrasonography for confirmation of gastric tube placement, concluding that there is insufficient evidence of the accuracy of ultrasonography for the confirmation of correct NGT placement as a single test, but intubated patients were excluded [18]. In a recent meta-analysis, due to heterogeneity of the studies, the diagnostic performance of ultrasonography was not straightforward, and the analysis concluded that chest X-rays should supplement the evaluation in case the ultrasonography is non-diagnostic 6]. In our study there was a high sensitivity in ultrasonographic evaluation (although the chest X-ray was not performed after every postural change), and the specificity was rather low, as there were seven patients in whom the NGT could not be identified with radiography, although u/s was informative and final confirmation came after aspiration of gastric fluid. Chest X-rays are not always diagnostic in the ICU; they are performed with a portable machine, anteroposterior, and in obese or edematous patients, the structures are not always well identified. Thus, ultrasonography may be superior to the gold standard for NGT correct placement.

Mumoli et al. evaluated 526 patients using bedside abdominal ultrasound to confirm correct NGT placement. Successful confirmation with ultrasonography was noted in 78.9% of the patients. The agreement between ultrasonographic confirmation and chest radiography was 0.94 (95% CI 0.91–0.96). Yet the study participants were a mixed population of patients with various degrees of impairment (or not) in the level of consciousness. The authors reported that when they excluded patients with altered mental status, the accuracy of the ultrasound technique slightly improved [19]. In our study, including only deeply sedated patients with COVID-19 ARDS, successful confirmation of the NGT was achieved in 89% of the screened patients. However, confirmation was not always evaluated using chest X-rays. Correct NGT placement was considered in cases where both ultrasonographic tests were present or where there was one ultrasonographic test and a positive palpation test.

In the patients presented, additional chest X-rays did not accompany every postural change to check for NGT correct position, and this may be a limitation, as the accuracy of the procedure was not confirmed formally. On the other hand, confirmation was performed in some cases, namely whenever an X-ray was ordered. The sensitivity of the technique was 98.9%, while specificity was only 57.9%, mainly due to difficulties in identifying the correct NGT placement through the chest X-ray. This raises the issue of the utility of chest X-ray as the gold standard method for the confirmation of correct NGT position. The current report is a presentation of our experience in a busy ICU, overwhelmed with COVID-19 ARDS patients, during a pandemic period. With this report we want to emphasize the value of POCUS in the COVID-19 era, expanding previous knowledge obtained from nine COVID-19 ARDS patients [20], and the potential to be used by critical care nurses as well. In this regard, with only a limited duration of training, accompanied with a small number of supervised examinations, critical care nurses can gain the skills required for POCUS. Although POCUS has been widely used in the critical care setting, becoming the new “stethoscope” for physicians, only scarce reports have arisen concerning its use by critical care nurses [21,22]. In depth learning of POCUS applicability should make it an invaluable tool for nurses as well. Another limitation of the study could be that other ultrasonographic windows to confirm correct NGT placement, such as the esophageal window in the neck, were not used, but this technique cannot exclude the misposition of the NGT in the esophagus [23].

In conclusion, POCUS can greatly improve everyday clinical practice for critical care staff, physicians, and nurses concerning the confirmation of correct nasogastric tube placement. It seems that in the settings where X-ray is not readily available, ultrasound may be useful to detect misplaced gastric tubes. Larger studies are needed to determine the possibility of adverse events when ultrasound is used to confirm nasogastric tube placement. Further studies on POCUS may unravel potential advantages of its use among physicians, nurses, and perhaps physiotherapists.

## Figures and Tables

**Table 1:** Baseline characteristics and main outcomes.

Characteristics	Patients
Age (mean ± SD), years	65.86 ± 12.29
Women (*n*, %)	111/276 (40.2%)
Ultrasonographic confirmation of NGT placement on ICU	246/276 (89.13%)
Ultrasonographic NGT visualization (on admission)	189/246 (76.8%)
Ultrasonographic whoosh test (on admission)	172/246 (69.9%)
Both NGT confirmation tests (on admission)	164/246 (66.7%)
Total number of ultrasonographic NGT confirmation tests (initial evaluation and re-evaluation)	590
Number of ultrasonographic NGT confirmation tests per patient	2.33 ± 1.23
Patients with four u/s examinations	45/246 (18.3%)
Patients with three u/s examinations	47/246 (19.1%)
Patients with two u/s examinations	44/246 (17.9%)
Patients with one u/s examinations	92/246 (37.4%)

ICU, Intensive Care Medicine; NGT, nasogastric tube; SD, Standard Deviation; u/s, ultrasound.

## Data Availability

All data are available upon request.

## Supplementary Materials

The following supporting information can be downloaded at https://www.mdpi.com/article/10.3390/jpm12030337/s1: Videos S1 and S2. Ultrasonographic whoosh test. A flash is seen in the center of the image, corresponding to the amount of air pushed through the nasogastric tube with a syringe. In Video S1, note that the image is vague (structures beneath, after instillation of the air, are not visualization, as the ultrasound beam does not penetrate the air).
